# Supplementary material for: Transient Increases in Inflammation and Proapoptotic Potential Are Associated with the HESN Phenotype Observed in a Subgroup of Kenyan Female Sex Workers
Source: Viruses. 2022 Feb 25;14(3):471. doi: 10.3390/v14030471 (PMC8948937; doi:10.3390/v14030471)
Supplement: Supplementary file 1 [file viruses-14-00471-s001.zip › viruses-1561549-supplementary.pdf]

Supplementary Figures and Tables:

Supplementary Table S1: Validated ISG Primer Pairs

Gene	Forward Primer Sequence 5'-3'	Reverse Primer Sequence 5'-3'
18s rRNA	Hs99999901_s1 (18S) FAM-MGB/S, ThermoFisher Scientific	
5LO	CACATGTTCCAGTCTTCTTGGA	ATGACCCGCTCAGAAATAGTGT
ACTB	ACT GGG ACG ACA TGG AGA AAA	GCC ACA CGC AGC TCA TTG TA
ANKRD1	GACCTCAACGCCAAAGACAGA	GGTTCAGTCTCACCGCATCA
APOBEC3F	GCA CCG CAC GCT AAA GGA	TTT TAA AGT GGA AGT AGA ATA TGT GTG GAT
ATM	CAGCAGCTGTTACCTGTTTG	TAGATAGGCCAGCATTGGAT
BAX	GATGCGTCCACCAAGAAGCT	CGGCCCCAGTTGAAGTTG
BCL2	CATGTGTGTGGAGAGCGTCAA	GCCGGTTCAGGTACTCAGTCA
BclXL/BCL2L1	GGTCGCATTGTGGCCTTT	TCCTTGTCTACGCTTTCCACG
BRCA1	CAG AGG ACA ATG GCT TCC ATG	CTA CAC TGT CCA ACA CCC ACT CTC
C5	GCGAGCTGCACGGATTAGTT	TGCGACGACACAACATTCAGT
CAR	AGCCTTCAGGTGCGAGATGTTACG	TACGACAGCAAAAGATGATAAGAC
CASP8	GTCTGTACCTTTCTGGCGGA	TCCAGTTTGCATTTGGAGATT
CASP9	GTTTGAGGACCTTCGACCAGCT	CAACGTACCAGGAGCCACTCTT
CCL5/RANTES	TAC ACC AGT GGC AAG TGC TC	GAA GCC TCC CAA GCT AGG AC
CDC25A	CAAACCTTGACAACCGATGC	ACACTGACCGAGTGCTGGAG
CDC25B	GGG CAA GTT CAG CAA CAT CGT GGA	GTA GCC GCC TTT CAG GAT ATA CAT C
CDK1	TGGATCTGAAGAAATACTTGGATTCTA	CAATCCCCTGTAGGATTTGG
CDK2	Tn GCT GAG ATG GTG ACT CG	GTA ACT CCT GGC CAe ACC AC
CDK4	TCC cn GAT CTG AGAATG GC	GAT CAC GGG CCT TGT ACA CT
CDK4A	AAATCTTTGACCTGATTGGG	CCTTATGTAGATAAGAGTGCTG
CDK7	ATG GCT CTG GAC GTG AAG TC	CIT AAT GGC GAC AAT ITG GIT G
CDK6	CTG CAG GGA AAG AAA AGT GC	CTC GAA GCG AAG TCC TCA AC

CDKN2A/p16/p14	CCCTCGTGCTGATGCTACTG	CATCATGACCTGGTCTTCTAGGAA
CIAP2	TGT TGG GAA TCT GGA GAT GA	CGG ATG AAC TCC TGT CCT TT
CXCL10/IP-10	TTC CTG CAA GCC AAT TTT GTC	TCT TCT CAC CCT TCT TTT TCA TTG T
DPP4/CD26	PrimePCR™ Template for SYBR® Green Assay: DPP4, Human, Bio-Rad Canada	
E2F1	CACAGATCCCAGCCAGTCTCTA	GAGAAGTCCTCCCGCACATG
EIF2AK2/PKR	CGT TGC TTA TGA ATG GTC TCA G	AGG AAG GTC AAA TCT GGG TG
FAS	AAA GTG GCC CAT ITA ACA GGC	AAA GCA GGA CAA ITC CAT AGG TG
FASL	AAAGTGGCCCATTTAACAGGC	AAAGCAGGACAATTCCATAGGTG
FCGR2B	PrimePCR™ Template for SYBR® Green Assay: FCGR2B, Human, Bio-Rad Canada	
FLAP-1/LRRFIP1	gagagacttccgacaccctcaa	cacctccacttcactggctctt
FLIP	GTG GAG ACC CAC CTG CTC A	GGA CAC ATC AGA TIT ATC CAA ATC C
GADD45	TCA GCG CAC GAT CAC TGT C	CCA GCA GGC ACA ACA CCA C
GPT2	TGAGCAATTCAGCCGAGAGA	GGTCTTCCGTCAGCTTTGCTT
HDM2	ACCCTGGTTAGACCAAAGCC	ACAATATGTTGTTGCTTCTCATCA
IDO1	AGA AGT GGG CTT TGC TCT GC	TGG CAA GAC CTT ACG GAC ATC TC
IFI6/G1P3	CCACCATGCGGCAGAAG	AAGTGAAGAGCAGCAGGTAGCA
IFIH1/MDA5	TClGTCGAGCCAGAGCTGAT	ACTCCTGAACCACTGTGAGCAA
IFIT1/ISG56	CAA CCA AGC AAA TGT GAG GA	AGG GGA AGC AAA GAA AAT GG
IFIT3/ISG60	TCC ACA CCA AAC AAT GGC TA	TGT TGC TIT TCA GCA TCA GG
IFNa1/13	tagacaaattctgcaccgaac	agatggagtccicattcatc
IFNA2	ggtagcaggaggaccttgatg	ggaggacagggatggtttcag
IFNA4	GAG GCC GAA GTI CAA GGT TA	TAA AGA AAA GGA CAG GGC CA
IFNB1	TTGTGCTTCTCCACTACAGC	CTGTAAGTCTGTTAATGAAG
IL-15	AAC AGA AGC CAA CTG GGT GAA TG	CTC CAA GAG AAA GCA CTT CAT TGC
IL-23 p19	ACACATGGATCTAAGAGAAGAGG	CTATCAGGGAGCAGAGAAGG
IL-28	CAGCTGCAGGTGAGGGA	GGTGGCCTCCAGAACCTT
IL-29	GGACGCCTTGGAAGAGTCACT	AGAAGCCTCAGGTCCCAATTC

IL-29	GGACGCCTTGGAAGAGTCACT	AGAAGCCTCAGGTCCCAATTC
IL-6	GGA GAC TTG CCT GGT GAA AA	ATC TGA GGT GCC CAT GCT AC
IL1B	ATC GGC CTC TAT TTG AAG ATA TGA CT	CCT CTA GGC TGG CTA TCT TTA TAC ATA CT
IRF-1	AAAAGGAGCCAGATCCCAAGA	CATCCGGTACACTCGCACAG
IRF-4	AGCAGTTCTTGTCAGAGC	GTTCTACGTGAGCTGTGATG
IRF-5	ATGCTGCCTCTGACCGA	GCCGAAGAGTTCCACCTG
IRF-7	GAG CCC TTA CCT CCC CTG TTA T	CCA CTG CAG CCC CTC ATA G
IRF-9	TGAGCCACAGGAAGTTACA	GAGCAGCAGTGAGTAGTCT
ISG15	AGC TCC ATG TGG GTG TCA G	GAA GGT CAG CCA GAA CAG GT
ISG20	AGATCCTGCAGCTCCTGAAA	TGTTCTGGATGCTCTTGTGC
LMP10	TCTGGGCGCCGATACG	TGGATCTTCTCGCAGCTCTTG
LMP7	TTACCTGCTTGGCACCATGTC	AGCAGGCGCTCCCAGTACT
MCL1	CGTTGTCTCGAGTGATGATCCA	ATTCCTGATGCCACCTTCTA
MXA	GGT GTC GAC ATA CCG GAA GA	CTC err GCA TGA GAG CAG TG
MxB	PrimePCR™ Template for SYBR® Green Assay: MX2, Human, Bio-Rad Canada	
MYD88	GAGCTGGCGGGCATCAC	TCGAAACGCTCAGGCATATG
OAS1	CCT GGT TGT cn CCT CAG TCC	GTG GAG AAC TCG CCC TCT n
OAS2	TGA GAG CAA TGG GAA ATG GG	AGG TAT TCC TGG ATA AAC CAA CCC
OASL	GGA TCT CCC ACA CTC ACA TCT	CAC CAT CAG GAT TCT TCA CGA A
P21cip1	CCT CAT CCC GTG TIC TCC TTl	GTA CCA CCC AGC GGA CAA GT
p53	TCA ACA AGA TGT TIT GCC AAC TG	ATG TGC TGT GAC TGC TTG TAG ATG
P8	TCTATAGCCTGGCCCATTCCT	TCTCTTGGTGCGACCTTTCC
RELA	AGCGCATCCAGACCAACAA	TAGTCCCCACGCTGCTCTTC
PELI1	TCAGGCTGCAAAGGCAATAAG	TCTGGGCCCGAGACAAAGTA
PMAIP1/NOXA	AGC TGG AAG TCG AGT GTG CT	TCC TGA GCA GAA GAG TIT GGA
PML / TRIM19	TGACCAGCATCTACTGCCG	AGCTCACTGTGGCTGCTGTC
PSME2	AGCTTTCCAGACAACCATTTCC	GGCCTTGGCCACAGCAT
PTGS2/COX2	GCTGGAACATGGAATTACCC	CTTTCTGTACTGCGGGTGGAA

	A	
PUMA	CCTGGAGGGTCCTGTACAATCT	GCACCTAATTGGGCTCCATCT
RB1	ACCTCAAACAAGGAAGAGAAATGAG	ACATCTGTGAGAGACAATGAATCCA
RIGI/DDX58	GCAGAGGCCGGCATGAC	TGTAGGTAGGGTCCAGGGTCTTC
RNASEL	GAC ACC TCT GCA TAA CGC AGT	GCG TGG CCC CAT TCT T
SOCS-1	TTTTCGCCCTTAGCGTGAAG	CATCCAGGTGAAAGCGGC
STAT1	CCT GCT GCG GTT CAG TGA	TCC ACC CAT GTG AAT GTG ATG
TAP1	TCCTGGTGGTCCTCTCCTCTCTT	GTGTTGTTATAGATCCCGTCACCC
TNF/TNFA	TCA ACC TCC TCT CTG CCA TC	CCT AAG CCC CCA ATT CTC TT
TNFRSF10A/TRAIL R1	CTGATGAAATGGGTCAACAA	AACTTTCCAGAGTCCACCAA
TNFSF10/TRAIL	GAGCTGAAGCAGATGCAGGAC	TGACGGAGTTGCCACTTGACT
TREX1	GGT GCC TTC TGT GTG GAT AGC	CTT CCT TGG GCC GTG TTC TG
TRIM11	CTA CGA GGC TGG ACA TCT CTC	CCG GCA GAT AGT CAT CGG G
TRIM25	CGGGTCGTGCCTGAATGAG	GCACCGTGTTCTTGTGCAG
TRIM32	TGT CCC TTT TGC AGC AAG ATT	GAT CTT TAG CAC TGT CAG ATT GTC TGT
TRIM5a	CTGGGTTGATGTGACAGTGG	ACACATTGCATCAGGTTGGA
Viperin/RSAD2	CAC AAA GAA GTG TCC TGC TTG GT	AAG CGC ATA TAT TTC ATC CAG AAT AAG
XIAPRF	GCT TGC AAG AGC TGG A TT IT	CAC TGG GCT TCC AAT CAG IT
YLPM1/ZAP	CCC GAG GGA ACT GTC GTT TT	GAT GGC CAG CAC CTT TCT GT
ZFP36L2	TGCGGGATCCAGAAACATG	GCACAAGAAGTCGACATCGTAGA
CX3CL1	CCTGTAGCTTTGCTCATCCACTATC	TCCAAGATGATTGCGCGTT
